# Clinical Characteristics and Treatment Outcomes of Oral Cancers Using Transoral Robotic Surgery in an Endemic Region

**DOI:** 10.3390/cancers15194896

**Published:** 2023-10-09

**Authors:** Chia-Chun Chang, Chung-Hsiung Chen, Tsai-Ling Hsieh, Kuang-Hsi Chang, Jing-Yang Huang, Frank Cheau-Feng Lin, Stella Chin-Shaw Tsai

**Affiliations:** 1 Department of Otolaryngology, Tungs’ Taichung MetroHarbor Hospital, Taichung 43503, Taiwan; t13154@ms3.sltung.com.tw (C.-C.C.); t5387@ms3.sltung.com.tw (C.-H.C.); t14159@ms3.sltung.com.tw (T.-L.H.); 2 Department of Medical Research, Tungs’ Taichung MetroHarbor Hospital, Taichung 43503, Taiwan; t12349@ms3.sltung.com.tw; 3 Jenteh Junior College of Medicine, Nursing and Management, Miaoli 35664, Taiwan; 4 Department of Medical Research, Chung Shan Medical University Hospital, Taichung 40201, Taiwan; cshe961@csh.org.tw; 5 Institute of Medicine, Chung Shan Medical University, Taichung 40201, Taiwan; 6 Department of Surgery, Chung Shan Medical University Hospital, Taichung 40201, Taiwan; 7 School of Medicine, Chung Shan Medical University, Taichung 40201, Taiwan; 8 Superintendents’ Office, Tungs’ Taichung MetroHarbor Hospital, Taichung 43503, Taiwan; 9 Department of Post-Baccalaureate Medicine, College of Medicine, National Chung Hsing University, Taichung 40227, Taiwan

**Keywords:** cancer registries, oral cancer, open surgery, overall survival, progression-free survival, propensity-score matching, transoral robotic surgery, minimally invasive surgery

## Abstract

**Simple Summary:**

Oral cancer is a significant health problem in Taiwan, with high rates of occurrence and cancer-related deaths. Transoral robotic surgery (TORS) is an innovative surgical approach that might offer treatment advantages and improve outcomes. We conducted a study in a Taiwanese hospital with 72 patients in each group, comparing TORS to conventional open surgery over three years. After carefully matching the patients, we found that those who had TORS had a better overall survival compared to the open-surgery group. We used various analyses to confirm that TORS was linked to a lower risk of death. The TORS group had significantly better survival rates at all stages of cancer, and it was particularly effective for late stages, i.e., stage III and IV patients. In conclusion, our study suggests that TORS could be a better option for treating oral cancer in Taiwan, offering improved chances of survival and potential advantages over conventional surgery.

**Abstract:**

Oral cancer poses a major health challenge in Taiwan, consistently ranking among the highest globally in both incidence and cancer-related mortality. Transoral robotic surgery (TORS) has potential advantages over open surgery, but its long-term oncologic outcomes are not well established. In this study, we sought to elucidate the role of TORS in improving treatment outcomes among oral cancer patients. A case–control study with propensity score matching was conducted in a single teaching hospital in Taiwan. It included 72 oral cancer patients in each group to analyze and compare survival outcomes between the surgical approaches. The TORS group demonstrated a higher negative resection margin rate, a lower mortality risk and better overall survival than the open-surgery group. Multivariate Cox regression analysis confirmed TORS’s association with a reduced risk of death. Kaplan–Meier survival analysis and log-rank tests indicated significantly better survival outcomes for the TORS group across all cancer stages. Moreover, the TORS group exhibited improved overall survival rates for stage III and IV patients compared to the conventional open-surgery group. In conclusion, this study suggests that TORS may offer better overall survival rates and potential advantages over conventional surgery for oral cancer treatment.

## 1. Introduction

Oral cancer is a major global health concern, exerting a profound impact on a vast number of individuals. According to the International Agency for Research on Cancer (IARC), oral cancer ranked as the 13th most prevalent cancer worldwide, with approximately 377,713 new cases and 177,757 deaths attributed to oral cancer in 2020 [1,2]. Within the specific context of Taiwan, oral cancer also poses a significant health burden. Among countries worldwide, Taiwan has one of the highest incidence rates of oral cancer, making it the fourth leading cause of cancer-related deaths [3,4]. Being endemic in Taiwan, oral cancer has a significantly higher prevalence among younger males in Taiwan. The incidence of oral cancer in Taiwan is associated with various risk factors, including the widespread use of tobacco, betel quid chewing, alcohol consumption, and defined genetic alterations [5,6].

As a malignant disease that adversely affects crucial functions such as chewing, speech, and swallowing, oral cancer also exerts significant social and psychological impacts by altering the individual’s appearance [5,7,8,9,10,11]. Therefore, selecting a surgical approach for oral cancer treatment is a critical decision that directly influences patient outcomes and long-term survival. In the era of powered instrumentation, imaging advances, and emerging surgical technology, transoral robotic surgery (TORS) is executed with precision and dexterity via the natural oral orifice, which is utilized in head and neck oncologic surgeries to achieve minimally invasive or even scar-less surgery, reducing the need for extensive incisions and associated morbidities of open surgery [12,13,14,15]. TORS offers potential advantages, such as improved preservation of organ function, enhanced cosmetic outcomes, reduced postoperative complications, and shorter hospital stays [16,17,18,19]. 

Despite these promising features, the comparative effectiveness of TORS versus conventional open surgery for oral cancer remains an area of ongoing debate and investigation. Limited evidence exists regarding the long-term oncological treatment outcomes, such as progression-free survival (PFS) and overall survival (OS) associated with TORS when compared to open surgery [12,20,21]. Hence, there is a critical need for further research to address this knowledge gap and provide a comprehensive understanding of the comparative effectiveness of TORS in improving survival rates and long-term oncologic outcomes for patients with oral cancer.

In this study, we aimed to contribute to the existing literature by conducting a retrospective analysis of a well-defined study population in Taiwan. Our study evaluated the comparative effectiveness of TORS versus conventional open surgery in terms of PFS and OS outcomes in patients with oral cancer. Through rigorous data collection, statistical analysis, and survival modeling, we aimed to provide valuable insights that can guide clinical decision making and optimize treatment strategies for patients with oral cancer.

## 2. Materials and Methods

### 2.1. Study Subjects

The study population consisted of consecutive patients who underwent initial oral cancer surgery at a regional teaching hospital from 1 January 2017 to 31 October 2021. Patients with cancers of the oral cavity (the International Classification of Diseases for Oncology, third edition, ICD-O-03: C00, C02, C03, C04, C050, C058, C059, and C06, excluding C024) were included. They were divided into two groups: the TORS and conventional open-surgery groups. The TORS group received transoral surgeries using da Vinci Si/Xi^®®^ (Intuitive Surgical, Sunnyvale, CA, USA) surgical system operated by a single surgeon. In contrast, the conventional open-surgery group received open surgeries by different surgeons, including the surgeon who performed TORS, other head and neck surgeons, and oral surgeons at a single institution. Propensity score matching was applied to match patients in a 1:1 ratio based on sex, age, primary site, and cancer stage, ensuring similar baseline characteristics between the two groups. Patients with a history of cancer, major head and neck surgeries, or unknown survival status were excluded from the analysis. Patients with oral cancer that extended to the oropharynx were included, but patients with cancers involving only the oropharynx, such as tonsils, soft palate, posterior pharyngeal wall, and tongue base, were excluded. The index date was defined as the first day of oral cancer surgery. The follow-up period spanned three years, until the patient withdrew, experienced disease progression, died, or the study concluded on 31 December 2022. Approval for this study was obtained from the Institutional Review Board of Tungs’ Taichung MetroHarbor Hospital (IRB number: 111067, date for the approval: 8 January 2022).

### 2.2. Data Collection

Data collection and assessment of survival outcomes in this study were conducted retrospectively using the hospital’s electronic medical records, the Tungs’ Hospital Cancer Registry, and the Taiwan Cancer Registry. The data collected for this study included the age, gender, primary site, cancer stage, margin status, other cancer treatment modalities such as concurrent chemoradiotherapy (CCRT), radiotherapy (RT), chemotherapy, target therapy (cetuximab), immunotherapy (nivolumab or pembrolizumab), and outcome measures such as disease progression and survival status. 

### 2.3. Statistical Analysis

This study used the chi-square test to compare categorical variables, presented as numbers (*n*) and percentages (%), and two-tailed t-tests to compare continuous variables, presented as mean ± standard deviation. To examine differences in disease-free survival (PFS) and overall survival (OS) between the TORS and conventional open-surgery groups, the Kaplan–Meier estimator was employed to analyze survival distributions for both surgery groups, and differences were compared using the two-sided log-rank test. Additionally, we used a Cox proportional hazards regression model to evaluate the effect of TORS on PFS and OS, using conventional open surgery as the reference and controlling for other potential baseline characteristics that may affect outcomes, such as gender, age, and other cancer treatments, and to calculate hazard ratios (HRs) and 95% confidence intervals (CIs) to assess statistical differences in outcomes. All statistical analyses were performed using SPSS 19.0 (SPSS Inc., Chicago, IL, USA). A two-tailed *p*-value less than 0.05 was considered statistically significant.

## 3. Results

### 3.1. Patient and Treatment Parameters in Oral Cancer Patients Treated with TORS versus Conventional Open Surgery

**Figure 1:**
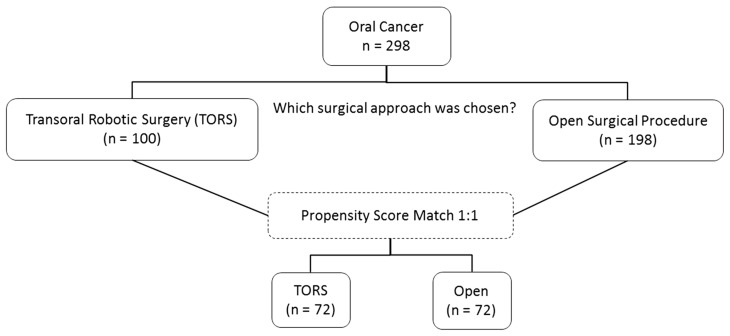
Sample selection diagram of the study participants.

A total of 298 consecutive patients with oral cancer were enrolled in this study, including 100 patients who underwent TORS and 198 patients who underwent open surgery. After employing a 1:1 matching technique using propensity score matching, 72 patients from each group were selected for analysis (Figure 1). 

The demographic and baseline characteristics of the patients are summarized in Table 1. The mean age of the participants was 57.8 ± 11.3 years, with the majority being males (94.4%). The most common primary site was the buccal area, followed by the tongue and gum, in both the open surgery and TORS groups. Based on the eighth edition of the AJCC TNM cancer staging manual, 48 patients (33.4%) were categorized as stage I or II, while 96 patients (66.7%) were classified as stage III or IV. The margin status exhibited a significantly more favorable outcome in the TORS group compared to the open-surgery group, with 97.2% versus 81.9% (*p* = 0.011) achieving negative margins, respectively.

### 3.2. Comparison of Survival Outcomes in Oral Cancer Patients Treated with TORS versus Conventional Open Surgery

During the three-year follow-up, the TORS group had an average PFS of 17.6 months (95% CI 13.9–21.2), while the open-surgery group had an average PFS of 15.8 months (95% CI 11.8–19.8). The TORS group also had a higher average OS of 29.82 months (95% CI 26.9–32.8) than the open-surgery group with an average OS of 21.16 months (95% CI 16.2–26.1). Although the surgical approach methods did not show a significant association with PFS in univariate analysis, the TORS group exhibited a lower OS risk than the open-surgery group (HR = 0.38, 95% CI 0.18–0.79, *p* = 0.009). Interestingly, the margin status of invasion was associated with significantly higher risks in PFS and OS in the univariate analysis (HR = 2.08 95% CI 1.09–3.97, *p* = 0.026 and HR = 3.05 95% CI 1.31–7.11, *p* = 0.010, respectively). In propensity-score-matched patients with oral cancer, CCRT, RT, and chemotherapy, which are common cancer treatment modalities, were linked to poorer PFS and an elevated risk of poor PFS (HR = 2.05, 95% CI = 1.29–3.26; HR = 2.13, 95% CI = 1.25–3.61; HR = 1.89, 95% CI = 1.04–3.43, respectively). Similarly, the utilization of RT, chemotherapy, target therapy, and immunotherapy were associated with an increased risk of death (HR = 3.82, 95% CI 1.34–10.92; HR = 4.32, 95% CI 1.03–18.08; HR = 2.62, 95% CI 1.31–5.26; HR = 3.23, 95% CI 1.49–6.98, respectively).

Furthermore, in a multivariate Cox regression analysis, while controlling for all associated factors, only the margin status of invasion was associated with a significantly worse PFS (HR = 1.99 95% CI 1.02–3.90, *p* = 0.044), while surgical approach method and target therapy demonstrated significant associations with the OS (Table 2). Specifically, TORS remained correlated with an improved OS (HR = 0.35, 95% CI 0.16–0.77, *p* = 0.009), whereas the utilization of target therapy increased the risk of death (HR = 2.27, 95% CI 1.07–4.84).

**Figure 2:**
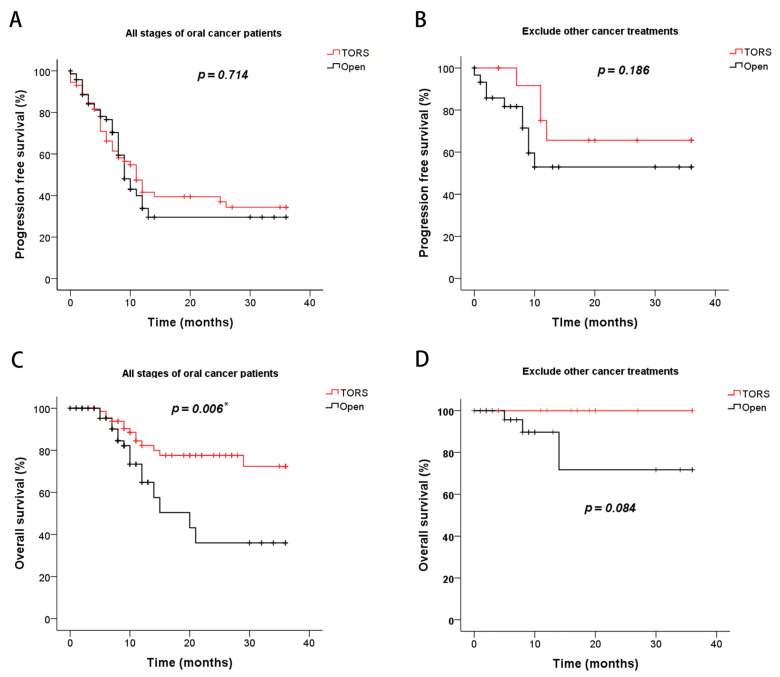
Kaplan–Meier curves for progression-free survival (PFS) and overall survival (OS) of oral cancer patients who underwent TORS and open surgery. (**A**) PFS of all patients; (**B**) PFS of patients, excluding those who received other cancer treatments; (**C**) OS of all patients; (**D**) OS of patients, excluding those who received other cancer treatments. Note: other cancer treatments received include chemotherapy, radiation, CCRT, target therapy, or immunotherapy. * *p* < 0.05 denotes statistical significance.

**Figure 3:**
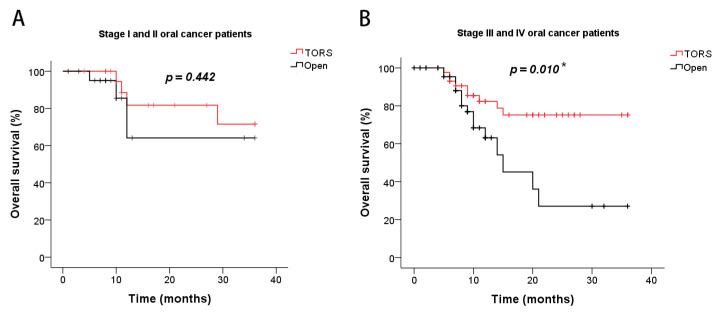
Kaplan–Meier analysis of overall survival for (**A**) stage I and II and (**B**) stage III and IV oral cancer patients in the TORS and open-surgery groups. * *p* < 0.05 denotes statistical significance.

The survival outcomes of PFS and OS were assessed using Kaplan–Meier survival analysis and two-sided log-rank tests. Regarding PFS, no significant difference was observed between the TORS and open-surgery groups in any stage of oral cancer (Figure 2A) or those patients who did not receive other cancer treatments (Figure 2B). However, regarding OS, the TORS group demonstrated a significantly better survival outcome than the open-surgery group among all patients regardless of cancer stage (*p* = 0.006, Figure 2C). There was no statistically significant difference in survival rates between the TORS and open-surgery groups when evaluating patients who underwent surgery alone without additional cancer treatments (Figure 2D). Nevertheless, it is worth noting that there was a noticeable trend in the separation of the PFS and OS curves when excluding other cancer treatments from the TORS versus open comparisons. Finally, in the analysis of cancer stage, explicitly distinguishing between early-stage (stage I and II) and advanced-stage disease (stages III and IV), the TORS group exhibited significantly improved OS for patients in stages III and IV compared to the open-surgery group (*p* = 0.010, Figure 3A,B).

## 4. Discussion

In this investigation, we found several pertinent findings highlighting the unique characteristics of oral cavity cancer in Taiwan and the potential advantages of robot-assisted surgery in this context. The results show that TORS was associated with a lower mortality risk and better OS than open surgery among all patients regardless of cancer stage (*p* = 0.006). Specifically, our results demonstrate that TORS showed better OS (HR = 0.34) and improved survival for stage III and IV patients. Even though no significant difference was found in PFS, except when excluding other treatments, TORS had a higher PFS (17.6 vs. 15.8 months) and OS (29.82 vs. 21.16 months) compared to open surgery. It is also worth highlighting that a notable trend separating the PFS and OS curves was observed when excluding other cancer treatments from the analysis. This suggests that the benefits of TORS in terms of survival outcomes may be more pronounced when not confounded by the influence of additional therapies.

Transoral robotic surgery provides clearer visualization of the operating field, allowing for precise wide excision of tumors that may be difficult to access, such as those located around corners obscured by dental structures, the tongue, mouth floor, deeper regions like the retropharyngeal margin, and even deeper extensions to the oropharynx [13,15,22]. The system’s better lighting, angled view, and three-dimensional assistance enhance surgical precision. The dexterity of endowrists in robot-assisted surgery even ensures tissue resection and enables 360-degree en bloc resection, contributing to optimal surgical outcomes. In contrast, within the conventional open-surgery group, transoral procedures might still be employed, especially for stage I and II patients, although all surgeries were performed under direct visual observation using only a headlight.

This study employed propensity score matching to compare the outcomes of TORS and open surgery in oral cancer patients [23]. During the matching process, certain oral cancer sites represented by ICD-O-03 codes C00 (lip), C04 (mouth floor), and C05 (other and unspecified parts of mouth) were excluded from the matching due to their limited occurrence and disparities in baseline characteristics between the TORS and open-surgery groups. Notwithstanding the exclusion of these specific oral cancer sites, the oral cancer sites chosen for this study effectively encompassed the most prevalent sites of oral cancers, both within the region under investigation and globally [3,5].

Importantly, oral cancer is a distinct disease entity in Taiwan, being different from oropharyngeal and hypopharyngeal cancers, as it is more radioresistant, and the majority of cases are not associated with human papillomavirus (HPV) [3,4]. Hence, surgery plays a significant role in treating oral cancers due to their unique characteristics and treatment requirements. The unique features of oral cavity cancer in Taiwan, such as low HPV positivity (33%) and its association with betel nut use (90%), present specific challenges in surgical management. Conditions like trismus, leukoplakia, and submucosal fibrosis can complicate surgery [24]. However, using robot-assisted surgery offers potential advantages in addressing these challenges [25,26,27,28].

Comparing the survival outcomes of TORS to open surgery, we observed better survival rates with the TORS approach. The use of a monopolar spatula in TORS may cause thermal injury but offers deeper cutting, improved hemostasis, and bleeding control, and facilitates en bloc resection of tumor [7,29]. Moreover, using an angled endoscope provides better visualization of critical vascular structures and nerves [30,31,32]. The inside-out extirpation of the tumor with TORS more closely resembles the natural course of tumorigenesis and invasion, reflecting the initiation and progression of oncological processes. The minimally invasive nature of robot-assisted surgery offers cosmetic advantages and functional preservation, contributing to improved patient outcomes [7,21,25,33,34].

In the innovative surgical approach to oral cancers using TORS, the lack of specific robot-assisted instruments for treating bone and teeth necessitates a hybrid approach involving the use of a robotic endoscope in combination with traditional cutting tools like saws, drills, and burrs for bony structures [35,36]. During such procedures, the operator may need to switch positions or rely on the assistance of the first assistant for cutting. Despite this limitation, the advantages of robot-assisted surgery outweigh the need for supplementary instruments and techniques, as demonstrated by our better survival outcomes.

Furthermore, this study examined the impact of various cancer treatment modalities on survival outcomes. Our study found that the utilization of CCRT, RT, target therapy, and immunotherapy was associated with worse survival outcomes. As propensity scores were utilized to mitigate the influence of staging factors, it is essential to consider that the need for additional treatments in some instances may indicate the presence of risk factors such as close or involved margins and extranodal extension. These factors could potentially explain the association between additional cancer treatments and poorer survival outcomes [5]. This highlights the complex nature of oral cancer treatment and underscores the need for a comprehensive approach that considers multiple factors to optimize patient outcomes [14,37,38,39,40].

Our study demonstrated that the utilization of the TORS surgical approach resulted in a significantly higher rate of achieving negative margins. The negative margin status was in turn associated with improved survival outcomes. In previous studies focused on TORS in HPV-related oropharyngeal cancers, it was indicated that a positive resection margin correlated with an increased risk of recurrences, either within the local region or as distant metastasis [27,41]. We propose that with the use of TORS, especially in advanced-stage oral cancer patients, better visualization and instrument dexterity might have helped to achieve negative resection margins and led to improved survival outcomes.

This study’s strengths include using a 1:1 matching technique to select comparable patient groups, utilizing reliable data sources such as electronic medical records and cancer registries, and including a three-year follow-up period. However, there are limitations to consider. Firstly, the retrospective nature of the study design introduces inherent biases and the potential for unmeasured confounders. Secondly, the study was conducted at a single hospital, which may limit the generalizability of the findings to other healthcare settings, but it had the advantage of including an open-surgery control group, providing valuable comparative data. Thirdly, our study lacked randomization, with patients requiring personal financial coverage for their TORS procedures. This financial factor could have introduced selection bias, potentially favoring individuals with greater economic resources. To counteract this potential bias, we diligently employed propensity score matching. Additionally, it is noteworthy that, given the Taiwan National Health Insurance’s non-coverage of often expensive treatments like target therapy and immunotherapy, we included these as variables in our multivariate analysis to account for their potential impact on the study outcomes.

## 5. Conclusions

This study provides valuable insights into the comparative outcomes of TORS versus open surgery for oral cancer. The results suggest that TORS is associated with achieving negative resection margins and improved overall survival rates in advanced-stage oral cancer patients and may offer advantages over traditional open surgery. These findings accentuate the importance of considering surgical approaches and other treatment modalities in managing oral cancer to optimize patient outcomes. Further prospective studies and randomized controlled trials are warranted to validate these findings and guide clinical decision making.

## Figures and Tables

**Table 1:** Patient demographic and baseline characteristics.

Characteristic	Open (*n* = 72)	TORS (*n* = 72)	Total (*n* = 144)	*p*-Value
Age, mean ± SD	58.6 ± 11.0	57.1 ± 11.5	57.8 ± 11.3	0.418
Gender, *n* (%)				
Male	69 (95.8%)	67 (93.1%)	136 (94.4%)	0.467
Female	3 (4.2%)	5 (6.9%)	8 (5.6%)	
Primary site (ICD 10), *n* (%)				
Tongue, dorsal (C020)	0 (0%)	1 (1.4%)	1 (0.7%)	0.775
Tongue, border (C021)	23 (31.9%)	17 (23.6%)	40 (27.8%)	
Tongue, ventral (C022)	2 (2.8%)	4 (5.6%)	6 (4.2%)	
Gum, upper (C030)	2 (2.8%)	2 (2.8%)	4 (2.8%)	
Gum, lower (C031)	10 (13.9%)	8 (11.1%)	18 (12.5%)	
Buccal (C060)	31 (43.1%)	34 (47.2%)	65 (45.1%)	
Retromolar area (C062)	4 (5.6%)	5 (6.9%)	9 (6.3%)	
Overlapping sites of mouth (C068)	0 (0%)	1 (1.4%)	1 (0.7%)	
Clinical T-stage, *n* (%)				
T1	14 (19.4%)	14 (19.4%)	28 (19.4%)	0.755
T2	24 (33.3%)	23 (31.9%)	47 (32.6%)	
T3	6 (8.3%)	10 (13.9%)	16 (11.1%)	
T4	28 (38.9%)	25 (34.7%)	53 (36.8%)	
Clinical N-stage, *n* (%)				
N0	40 (55.6%)	33 (45.8%)	73 (50.7%)	0.335
N1	10 (13.9%)	10 (13.9%)	20 (13.9%)	
N2	20 (27.8%)	25 (24.7%)	45 (31.3%)	
N3	2 (2.8%)	1 (1.4%)	3 (2.1%)	
Nx	0 (0%)	3 (4.2%)	3 (2.1%)	
Clinical disease stage, *n* (%)				
I	10 (13.9%)	11 (15.3%)	21 (14.6%)	0.987
II	13 (18.1%)	14 (19.4%)	27 (18.8%)	
III	9 (12.5%)	9 (12.5%)	18 (12.5%)	
IV	40 (55.6%)	38 (52.8%)	78 (54.2%)	
Margin status, *n* (%)				
Free	60 (81.9%)	70 (97.2%)	130 (89.6%)	0.011 *
Invasion	12 (16.7%)	2 (2.8%)	12 (9.7%)	
Unkown	1 (1.4%)	0 (0%)	1 (0.7%)	

* *p* < 0.05 denotes statistical significance.

**Table 2:** Univariate and multivariate analyses of progression-free survival and overall survival in oral cancer patients.

	Univariate				Multivariate			
	Progression-Free Survival		Overall Survival		Progression-Free Survival		Overall Survival	
Variable	HR (95%CI)	*p*-Value	HR (95%CI)	*p*-Value	HR (95%CI)	*p*-Value	HR (95%CI)	*p*-Value
Gender								
Female (ref.)	1.00		1.00		1.00		1.00	
Male	1.23 (0.45–3.36)	0.691	0.81 (0.19–3.37)	0.767	1.47 (0.51–4.23)	0.477	0.89 (0.19–4.14)	0.880
Age	1.00 (0.98–1.02)	0.701	1.01 (0.98–1.04)	0.450	1.00 (0.98–1.02)	0.872	1.02 (0.98–1.05)	0.315
Clinical disease stage								
I and II (ref.)	1.00		1.00					
III and IV	1.06 (0.65–1.72)	0.816	1.85 (0.8–4.29)	0.151				
Surgical approach								
Open (ref.)	1.00		1.00		1.00		1.00	
TORS	0.92 (0.59–1.45)	0.722	0.38 (0.18–0.79)	0.009 *	0.92 (0.57–1.47)	0.722	0.35 (0.16–0.77)	0.009 *
Margin status								
Free (ref.)	1.00		1.00		1.00		1.00	
Invasion	2.08 (1.09–3.97)	0.026 *	3.05 (1.31–7.11)	0.010 *	1.99 (1.02–3.90)	0.044 *	1.13 (0.41–3.15)	0.812
Unkown	0.00 (0.00–9.28)	0.967	0.00 (0.00–0.00)	0.986	0.00 (0.00–2.46)	0.970	0.00 (0.00–0.00)	0.982
CCRT								
Yes vs. No	2.05 (1.29–3.26)	0.002 *	1.91 (0.92–3.98)	0.082	1.57 (0.80–3.08)	0.192		
RT								
Yes vs. No	2.13 (1)	0.005 *	3.82 (1.34–10.92)	0.012 *	1.49 (0.69–3.25)	0.315	2.96 (0.93–9.43)	0.066
Chemotherapy								
Yes vs. No	1.89 (1.04–3.43)	0.036 *	4.32 (1.03–18.08)	0.045 *	1.09 (0.50–2.38)	0.836	1.34 (0.27–6.67)	0.721
Cetuximab								
Yes vs. No	1.45 (0.89–2.36)	0.140	2.62 (1.31–5.26)	0.007 *			2.27 (1.07–4.84)	0.034 *
Nivolumab/ Pembrolizumab								
Yes vs. No	1.79 (0.97–3.31)	0.065	3.23 (1.49–6.98)	0.003 *			2.23 (0.93–5.36)	0.072

CCRT: concurrent chemoradiotherapy; RT: radiotherapy; * *p* < 0.05 denotes statistical significance.

## Data Availability

The data presented in this study are available at https://doi.org/10.7910/DVN/BJYGUD.
